# Clavicle fractures: epidemiology, classification and treatment of 2 422 fractures in the Swedish Fracture Register; an observational study

**DOI:** 10.1186/s12891-017-1444-1

**Published:** 2017-02-15

**Authors:** Caroline Kihlström, Michael Möller, Katarina Lönn, Olof Wolf

**Affiliations:** 1 Department of Orthopaedics, Institute of Surgical Sciences, Uppsala University Hospital, SE-751 85 Uppsala, Sweden; 2 Department of Orthopaedics, Sahlgrenska University Hospital Gothenburg/ Mölndal, SE-431 80 Mölndal, Sweden

**Keywords:** Clavicle fracture, Epidemiology, Classification, Treatment, The Swedish Fracture Register

## Abstract

**Background:**

Large multi-centre studies of clavicle fractures have so far been missing. The aim of this observational study was to describe the epidemiology, classification and treatment of clavicle fractures in the The Swedish Fracture Register (SFR) that collects national prospective data from large fracture populations.

**Methods:**

Data were retrieved from the SFR on all clavicle fractures sustained by patients ≥ 15 years of age in 2013–2014 (*n* = 2 422) with regards to date of injury, cause of injury, fracture classification and treatment.

**Results:**

Sixty-eight per cent of the clavicle fractures occurred in males. The largest subgroup was males aged 15–24 years, representing 21% of clavicle fractures. At the ages of 65 years and above, females sustained more clavicle fractures than males. Same-level falls and bicycle accidents were the most common injury mechanisms. Displaced midshaft fractures constituted 43% of all fractures and were the most frequently operated fractures. Seventeen per cent of the patients underwent operative treatment within 30 days of the injury, where plate fixation was the choice of treatment in 94% of fractures.

**Conclusion:**

The largest patient group was young males. Displaced midshaft fractures were the most common type of clavicle fracture as well as the most frequently operated type of fracture.

## Background

Fractures of the clavicle, which primarily occur in young males, constitute 2.6–4% of all fractures in adults [1, 4]. A male dominance of approximately 70% has been reported [1–4]. The most frequent injury mechanism is a direct fall on the shoulder [2–4]. Fractures are often sustained during sports activities or traffic accidents [1–4].

The majority (69–82%) of fractures occur in the midshaft of the clavicle, followed by 12–26% in the lateral part and 2–6% in the medial part [1–4]. This can be anatomically explained by the fact that the medial and lateral parts of the clavicle are firmly secured by strong ligaments and muscles, whereas the middle part of the clavicle lacks any strong attachments and thus is more vulnerable to trauma. The muscle attachments often cause a dislocation of the major fragments in clavicle fractures and a shortening of the clavicle, particularly in midshaft fractures [5].

Traditionally, clavicle fractures have been treated almost exclusively non-operatively, regardless of the type of fracture. Studies in the 1960s described good functional results for non-operatively treated midshaft clavicle fractures and a lower nonunion rate compared to fractures treated with primary open reduction [6, 7]. In contrast, several more recent studies have reported opposite results with newer methods of fracture fixation [8–14], which may have contributed to the 705% increase in operative treatment of clavicle fractures in Sweden between 2001 and 2012 [15]. Optimal treatment of clavicle fractures however remains a debated subject.

Simple slings, collar ‘n’ cuffs and figure-of-eight-bandages are commonly used to immobilise the fracture during the first weeks in non-operatively treated fractures [16], which often include medial fractures, most lateral fractures and midshaft fractures without displacement [17].

The most commonly used operative method today is open reduction and internal plate fixation; a smaller number of fractures are treated with intramedullary nails, pins or wires [14, 17].

Because the treatment of clavicle fractures is a debated question and because there are no national guidelines in place for it in Sweden, treatment can vary between different departments, with regards both to which fractures are operated and operative method chosen ([18]).

Previous studies on clavicle fractures have generally been limited to one specific orthopaedic department at a time and thus to a limited patient material [2–4] with susceptibility to local treatment traditions that may not adequately reflect a more general treatment routine. Multi-centre studies that include conformed data from a large number of departments have so far been missing. The largest study populations in epidemiological studies are also at least 20 years old [2–4]. Much has happened in the area of treatment of clavicle fractures since then, especially with the rate of operative treatment having increased substantially even with an absence of studies showing compelling evidence to support this [15]. An updated study on the current epidemiology, classification and treatment of clavicle fractures in a more generalised setting would hopefully create a framework for contextual aid for future analysis of the best treatment for clavicle fractures. The aim of this study was therefore to describe the modern epidemiology, classification and treatment of clavicle fractures in Sweden, with a secondary aim of assessing the presence of polytrauma in patients with clavicle fractures.

## Methods

### Data collection and study population

In the Swedish Fracture Register (SFR), started in 2011, information about fractures of the extremities, pelvis and spine is registered locally in affiliated departments, building up a national database of the epidemiology, classification and treatment of different fractures [19]. Affiliated departments are hospital-based and include university hospitals, district general hospitals and general hospitals since 2013, creating a mixed catchment population. At the end of 2013, there were 7 registering departments (1 university hospital, 5 general hospitals and 1 district general hospital). The number had increased to 22 at the end of 2014 (3 university hospitals, 11 general district hospitals and 8 district hospitals). The number of orthopaedic departments that treat fractures in Sweden is approximately 55. Hence apprixmately 44% of Sweden’s orthopaedic departments reported to the SFR at the end of the study period. Due to lack of full national coverage, the SFR can not provide numbers on incidence of fractures but a recent study [15] showed that the incidence of clavicle fractures in Sweden increased from 35.6 per 100,000 person-years in 2001 to 59.3 per 100,000 person-years in 2012. Clavicle fractures have been registered since April 2012 [19–21].

Data collected in the SFR include date of injury, cause of injury, fracture classification and treatment. Inclusion requires the patient to have a permanent Swedish personal identity number and a radiographically verified fracture. Fractures need also to have occurred in Sweden. Fractures that have occurred abroad are excluded from registration [19]. Completeness of registrations of clavicle fractures has yet to be investigated. We have used data from the SFR to construct an observational descriptive register study, employing a cross-sectional design. Selection criteria were all registered clavicle fractures sustained in 2013–2014 and patients had to be at least 15 years of age. No additional exclusion criteria were applied.

Medical records and radiographs were also reviewed for the presence of polytrauma in a subset local population comprising all clavicle fractures that were treated at the authors’ own orthopaedic department at Uppsala University Hospital during the selected period. The selection of department was made so as to allow for full access to medical records and radiographs. This subpopulation was very similar to the overall population with regards to age, sex and fracture type distribution and is as such representative of the overall study population.

### Variables

**Fig. 1:**
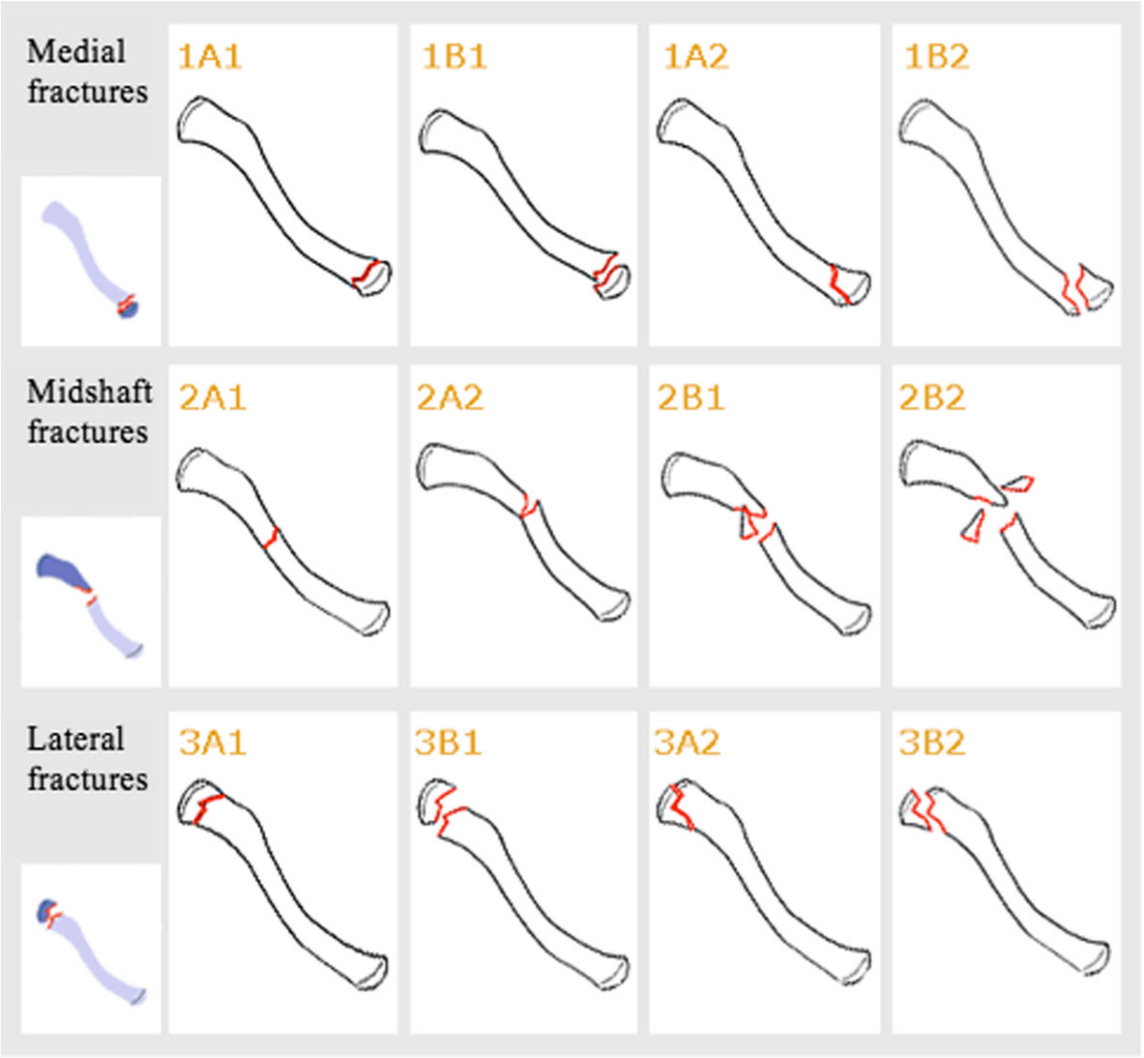
Robinson’s classification system for clavicle fractures as presented in the SFR’s online registration module (18). *1A1* Medial, undisplaced, extra-articular. *1B1* Medial, displaced, extra-articular. *1A2* Medial, undisplaced, intra-articular. *1B2* Medial, displaced, intra-articular. *2A1* Midshaft, cortical alignment, undisplaced. *2A2* Midshaft, cortical alignment, angulated. *2B1* Midshaft, displaced, simple or wedge comminuted. *2B2* Midshaft, displaced, isolated or comminuted segmental. *3A1* Lateral, cortical alignment, extra-articular. *3B1* Lateral, displaced, extra-articular. *3A2* Lateral, cortical alignment, intra-articular. *3B2* Lateral, displaced, intra-articular. The use of the figure in this study has been approved by the SFR
- Primary treatment
  Operative treatment methods were divided into fixation with anatomical plates, standard plates, hook plates, intramedullary fixations and other methods. Who performed the operation was also taken into account, i.e. a consultant orthopaedic trauma surgeon, a consultant orthopaedic surgeon or a resident. For the non-operatively treated patients no information was provided on the type of sling received for short-term immobilisation or on the application of physiotherapy.
  Operative treatment was divided into an acute stage and an early stage. Acute stage operations are defined as such when they are registered in the SFR as the first type of treatment for a particular fracture. Early operations are defined as operations where non-operative treatment was the first registered choice of treatment but was abandoned early on for secondary operative treatment, typically after an X-ray follow-up 7–10 days after the injury shows a worsened fracture position. An upper cut-off value of 30 days was applied to filter out seemingly faulty registrations. Fractures being treated operatively after more than 30 days post-injury were considered to have been treated non-operatively as the first choice of treatment.
- Polytrauma
  Polytrauma was defined as having multiple radiographically verified concurrent fractures in addition to a clavicle fracture. All fracture types except for rib fractures were included, because they are usually radiographically diagnosed only if a trauma computed tomography scan has been performed and typically not seen on clavicular projections with conventional x-rays, making their frequency difficult to assess.

- Injury mechanism
  Four main categories were constructed for injury mechanism – falls, transport accidents, non-traumatic fractures and others. Falls were further sub-categorised into falls on the same level, falls from a level and unspecified falls. Transport accidents were sub-categorised into bicycle accidents, motorcycle accidents and other transport accidents. Pathological fractures, spontaneous fractures and stress fractures were grouped together and labeled non-traumatic fractures. The other-category included patients who had sustained their clavicle fractures for example from having been pushed to the ground or having suffered a direct impact from a person or object. Many sporting injuries sort into this category.
  The registering doctor in the SFR classifies the energy level of the injury. The SFR has no strict guidelines for how to make the distinction between high- and low energy injuries, making the distinction rather subjective. Examples on how to classify the energy level are however presented in the registration module. Examples of high-energy injuries are traffic accidents, falls from heights and work place accidents with crushing injuries. Low-energy injuries are exemplified as falls on the same level and similar traumas.
- Fracture classification
  Clavicle fractures were classified according to Robinson’s classification system for registration in the SFR (Fig. 1) [3].

### Statistical analysis

Data was summarised for fracture occurence with groupings of sex, age with subgroupings of both 10-year intervals and groupings of young (15–24 year-olds), mature (25–64 year-olds) and old (over 65 year-olds), time of year and day of the week. Because of the descriptive nature of the study, formal testing of potential differences between subgroups was not made. Calculations of means, first and third quartiles and standard deviations (SD) were made.

A minority of the registrations (4%, *n* = 87) were incomplete and lacked one or more types of particular data, such as injury mechanism (1%, *n* = 24), energy level of injury (1%, *n* = 33) or type of treatment (2%, *n* = 41). In these cases data analysis of percentages is based on the total of each completely registered sub-data set.

## Results

### Epidemiology

**Fig. 2:**
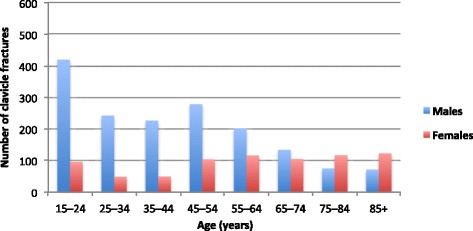
Distribution of clavicle fractures by age and sex

We found 2 422 registered clavicle fractures in 2013–2014; 1 056 in 2013, 1 366 in 2014. Sixty-eight per cent (*n* = 1 654) of the clavicle fractures occurred in males and 32% (768) in females, creating a male:female ratio of 2.2:1. Mean age was 48 years (SD 23 years). Mean age was higher in females (mean 59 years, SD 23 years) than in males (mean 43 years, SD 21 years). The fractures occurred more often in younger than in older individuals (Fig. 2), with 15–24 year-olds representing 21% (*n* = 517) of the study population. Males in this age group represented 17% (*n* = 420) of the total fracture burden. As many as 45% of the females (*n* = 348) but only 17% (*n* =283) of the males were 65 years or older, creating a male:female ratio of 0.8:1 (*n* = 283:348) within the age group.

**Fig. 3:**
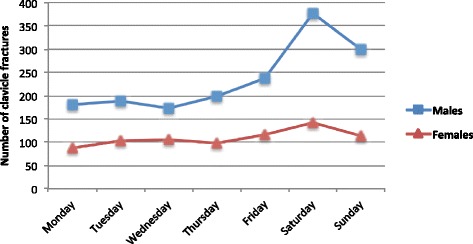
Daily distribution of clavicle fractures by sex

**Fig. 4:**
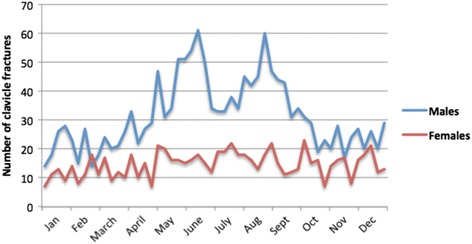
Monthly distribution of clavicle fractures by sex

Fractures more frequently occurred during weekends, particularly on Saturdays, and had a peak occurrence in the summer months of May to August (Figs. 3 and 4).

### Injury mechanism

The most common cause of injury was either a fall, generally on the same level, or a transport accident (Table 1). Bicycle accidents were by far the most common cause among the transport accidents, followed by motorcycle accidents. Males and younger patients most commonly sustained their clavicle fractures from transport accidents in comparison to females and older patients who more often sustained their clavicle fractures from a fall.

**Table 1:** Injury mechanism by sex, age and high-energy injuries

	Total, *n* (%)	Males, *n* (%)	Females, *n* (%)	Young, *n* (%)	Mature, *n* (%)	Old, *n* (%)	High-energy (%)
**Fall**	**1 175 (49.0)**	**663 (40.5)**	**512 (67.5)**	**200 (39.0)**	**467 (37.0)**	**508 (81.5)**	**101 (15.2)**
Fall on the same level	817 (34.1)	469 (28.6)	348 (45.8)	169 (32.9)	303 (24.0)	345 (55.4)	41 (6.2)
Fall from a height	210 (8.8)	117 (7.1)	93 (12.3)	10 (1.9)	101 (8.0)	99 (15.9)	55 (8.3)
Unspecified fall	148 (6.2)	77 (4.7)	71 (9.4)	21 (4.1)	63 (5.0)	64 (10.3)	5 (0.8)
**Transport accident**	**996 (41.5)**	**803 (49.0)**	**193 (25.4)**	**203 (39.6)**	**705 (55.9)**	**88 (14.1)**	**539 (81.1)**
Bicycle	492 (20.5)	404 (24.6)	88 (11.6)	58 (11.3)	394 (31.2)	40 (6.4)	183 (27.5)
Motorcycle	309 (12.9)	298 (18.2)	11 (1.4)	95 (18.5)	198 (15.7)	16 (2.6)	233 (35.0)
Other transport accident	195 (8.1)	101 (6.2)	94 (12.4)	50 (9.7)	113 (9.0)	32 (5.1)	123 (18.5)
**Non-traumatic fractures**	**17 (0.7)**	**7 (0.4)**	**10 (1.3)**	**0 (0)**	**6 (0.5)**	**11 (1.8)**	**0 (0)**
**Other**	**210 (8.8)**	**166 (10.1)**	**44 (5.8)**	**110 (21.4)**	**84 (6.7)**	**16 (2.6)**	**25 (3.8)**
**Total**	**2 398**	**1 639**	**759**	**513**	**1 262**	**623**	**665**

Young = 15–24 years of age, Mature = 25–64 years old, Old = 65+ years of age﻿. Main groups of injury mechanisms are presented in bold letters, followed by subgroups in normal letters

High-energy trauma was reported as the type of injury in 28% (*n* = 668) of the fractures. Males sustained more high-energy injuries than females: males 35% (*n* = 538) versus females 17% (*n* = 130). The mean age for high-energy injuries was also lower (41 years, SD 18 years) than that for low-energy injuries (51 years, SD 24 years).

Non-traumatic fractures included pathological fractures (*n* = 10), spontaneous fractures (*n* = 5) and stress fractures (*n* = 2).

### Fracture classification

Fifty-two per cent (*n* = 1 271) of the clavicle fractures occurred on the left side. Four patients sustained simultaneous bilateral fractures and another 11 sustained multiple clavicle fractures on the same or opposite side at separate times of injury during the 2-year period. Only 0.7% (*n* = 16) of clavicle fractures were open fractures.

The most frequent fracture location was the midshaft of the clavicle. Among the midshaft fractures, 90% (*n* = 1 424) had some type of angulation or displacement (2A2, 2B1, 2B2) (Table 2). The most common fractures of all were the midshaft simple displaced or wedge comminuted 2B1 fractures. Medial fractures were uncommon. Ninety per cent (*n* = 649) of the lateral fractures were extra-articular. Lateral fractures were slightly more often displaced than undisplaced.

**Table 2:** Fracture classification by sex, age and high-energy injuries

	Total, *n* (%)	Males, *n* (%)	Females, *n* (%)	Young, *n* (%)	Mature, *n* (%)	Old, *n* (%)	High energy, *n* (%)
**Medial**	**109 (4.5)**	**68 (4.1)**	**41 (5.3)**	**8 (1.5)**	**30 (2.4)**	**71 (11.3)**	**16 (2.4)**
1A1	49 (2.0)	33 (2.0)	16 (2.1)	4 (0.8)	15 (1.2)	30 (4.8)	7 (1.0)
1A2	16 (0.7)	9 (0.5)	7 (0.9)	1 (0.2)	5 (0.4)	10 (1.6)	3 (0.4)
1B1	33 (1.4)	18 (1.1)	15 (2.0)	3 (0.6)	7 (0.5)	23 (3.6)	3 (0.4)
1B2	11 (0.5)	8 (0.5)	3 (0.4)	0 (0)	3 (0.2)	8 (1.3)	3 (0.4)
**Midshaft**	**1 584 (65.4)**	**1 168 (70.6)**	**416 (54.2)**	**439 (84.9)**	**889 (69.8)**	**256 (40.6)**	**550 (82.3)**
2A1	160 (6.6)	113 (6.8)	47 (6.1)	62 (12.0)	70 (5.5)	28 (4.4)	50 (7.5)
2A2	380 (15.7)	276 (16.7)	104 (13.5)	161 (31.1)	158 (12.4)	61 (9.7)	107 (16.0)
2B1	676 (27.9)	489 (29.6)	187 (24.3)	161 (31.1)	387 (30.4)	128 (20.3)	232 (34.7)
2B2	368 (15.2)	290 (17.5)	78 (10.2)	55 (10.6)	274 (21.5)	39 (6.2)	161 (24.1)
**Lateral**	**719 (29.7)**	**410 (24.8)**	**309 (40.2)**	**68 (13.2)**	**351 (27.6)**	**300 (47.5)**	**100 (15.0)**
3A1	291 (12.0)	144 (8.7)	147 (19.1)	25 (4.8)	126 (9.9)	140 (22.2)	25 (3.7)
3A2	29 (1.2)	20 (1.2)	9 (1.2)	1 (0.2)	14 (1.1)	14 (2.2)	6 (0.9)
3B1	358 (14.8)	215 (13.0)	143 (18.6)	34 (6.6)	189 (14.8)	135 (21.4)	63 (9.4)
3B2	41 (1.7)	31 (1.9)	10 (1.3)	8 (1.5)	22 (1.7)	11 (1.7)	6 (0.9)
**Not classified**	**10 (0.4)**	**8 (0.5)**	**2 (0.3)**	**2 (0.4)**	**4 (0.3)**	**4 (0.6)**	**2 (0.3)**
**Total**	**2 422**	**1 654**	**768**	**517**	**1 274**	**631**	**668**

Young = 15–24 years of age, Mature = 25–64 years old, Old = 65+ years of age. Main groups of fracture classification are presented in bold letters, followed by subgroups in normal letters

Displaced midshaft fractures (2B1 and 2B2) were found in 47% (*n* = 779) of the male patients versus 35% (*n* = 265) of the female patients. Conversely, lateral fractures were more frequent in females than in males. Medial and lateral fractures were much more common in the higher age groups while younger patients typically sustained midshaft clavicle fractures.

The majority of the high-energy injuries resulted in displaced midshaft clavicle fractures.

### Primary treatment

Eleven per cent (*n* = 270) of all fractures were treated operatively in the acute stage as the first choice of treatment after a median of 5 days (interquartile range 4–10 days). An additional 6% (*n* = 138) of the fractures were treated operatively after non-operative treatment had been abandoned at an early stage, after a median of 14 days (interquartile range 11–17 days).

Males, in comparison with females, were more likely to undergo operative treatment in the acute or early stages: 20% (*n* = 323) of the males versus 11% (*n* = 85) of the females. The mean age for operative treatment was 36 years (SD 15 years). The mean age for non-operative treatment was 51 years (SD 23 years).

Eighty percent of the operatively treated fractures were midshaft fractures (Table 3). The most frequently operated fractures were the fully displaced 2B1 and 2B2 midshaft fractures. Together, these two fracture types accounted for 73% (*n* = 296/408) of the operatively treated fractures. A fair few of the lateral displaced 3B1 and 3B2 fractures were also treated operatively but since they were not very frequent to begin with, they accounted for less than 20% of the total number of operated fractures. Few of the midshaft and lateral fractures without full displacement (2A1, 2A2, 3A1, 3A2) and none of the medial fractures were treated operatively.

**Table 3:** Operatively treated clavicle fractures in an acute or early stage by fracture classification including operative method

	Total, *n* (%)	Anatomical plate, *n* (%)	Hook plate, *n* (%)	Standard plate, *n* (%)	Intramedullary fixation, *n* (%)	Other method, *n* (%)
**Midshaft**	**326 (79.9)**	**281 (88.6)**	**1 (2.4)**	**24 (96.0)**	**19 (100.0)**	**1 (20)**
2A1	2 (0.5)	2 (0.6)	0 (0)	0 (0)	0 (0)	0 (0)
2A2	26 (6.4)	20 (6.3)	0 (0)	3 (12.0)	3 (15.8)	0 (0)
2B1	158 (38.7)	134 (42.3)	0 (0)	11 (44.0)	13 (68.4)	0 (0)
2B2	140 (34.3)	125 (39.4)	1 (2.4)	10 (40.0)	3 (15.8)	1 (20)
**Lateral**	**82 (20.1)**	**36 (11.4)**	**41 (97.6)**	**1 (4.0)**	**0 (0)**	**4 (80.0)**
3A1	4 (1.0)	1 (0.3)	2 (4.8)	0 (0)	0 (0)	1 (20.0)
3A2	0 (0)	0 (0)	0 (0)	0 (0)	0 (0)	0 (0)
3B1	68 (16.7)	33 (10.4)	33 (78.6)	1 (4.0)	0 (0)	1 (20.0)
3B2	10 (2.5)	2 (0.6)	6 (14.3)	0 (0)	0 (0)	2 (40.0)
**Not classified**	**0 (0)**	**0 (0)**	**0 (0)**	**0 (0)**	**0 (0)**	**0 (0)**
**Total**	**408**	**317**	**42**	**25**	**19**	**5**

Medial fractures are not included in the table because none of the medial fractures were treated operatively. Main groups of fracture classification are presented in bold letters, followed by subgroups in normal letters

Anatomical plates were by far the most common choice of operative treatment. Hook plates were used mainly for the lateral displaced extra-articular 3B1 fractures while intramedullary nailing was used mainly for the angulated midshaft 2A2 and simple displaced or wedge comminuted 2B1 fractures.

Consultant orthopaedic trauma surgeons performed 56% (*n* = 227) of the operations, consultant orthopaedic surgeons 35% (*n* = 142) and residents 7% (*n* = 27) (2% of surgeons were undefined).

### Polytrauma

In the locally reviewed population of 321 clavicle fractures at Uppsala University Hospital 21% of patients (*n* = 66) had multiple radiographically verified concurrent fractures in addition to their clavicle fracture. The most prevalent concurrent fractures were those of the vertebral column, scapula, cranium and forearm (Table 4).

**Table 4:** Concurrent fractures within each fracture group in addition to a patient’s clavicle fracture in patients seen at Uppsala University Hospital

Concurrent fracture location	Fractures, *n* (%)
Vertebral column	19 (18)
Scapula (including 2 floating shoulders)	15 (15)
Cranium	13 (13)
Radius and/or ulna	11 (11)
Hand	10 (10)
Humerus	7 (7)
Sternum	7 (7)
Pelvis	6 (6)
Femur	5 (5)
Tibia and/or fibula	5 (5)
Contralateral clavicle, foot or patella	5 (5)
Total	103

## Discussion

### Main findings

In this observational study of clavicle fractures in Sweden, the largest patient group was males younger than 25 years of age and the most frequent causes of injury were same-level falls, bicycle and motorcycle accidents. Displaced midshaft fractures were the most common type of fracture. These fractures, together with extra-articular displaced lateral fractures, were also the most frequently operated fractures. Seventeen per cent of the fractures underwent operative treatment within 30 days of the injury, most commonly with plate fixation.

### Comparisons with other studies

The sex-related distribution of clavicle fractures is in line with that reported in previous studies [1–4]. The mean age of our population of 48 years is actually higher than several other studies of adults, where the mean age ranged from 29 to 34 years. However, the youngest age for inclusion has varied between these studies. As in previous studies, we found that the mean age was highest for fractures occurring in the medial part of the clavicle and lowest for midshaft fractures and that the mean age was higher in females than in males [1–4].

Clavicle fractures occurred more frequently during the weekends and during the summer months. The finding is consistent with previous Swedish studies [2, 4] but in contrast with the findings of an Italian study [1]. Inasmuch as clavicle fractures are closely related to physical activities, the difference in frequency is possibly due in large part to an increase in outdoor activities during summer and weekends in Sweden.

Same-level falls have been reported as the most common cause of clavicle fractures not only in this but in previous studies as well [2, 3]. An Italian study reported the most common cause of fractures to be accidental falls in elderly but traffic accidents in young adults [1], which is not far off from the results of this study.

The finding that bicycle accidents were the second most common cause of clavicle fractures is in agreement with another Swedish epidemiological study demonstrating that bicycle accidents caused 45% of the clavicle fractures in females and 26% in males aged 15 years and above [2]. It is however in contrast with the findings of a Scotland-based epidemiological study [3], where only 11% of clavicle fractures were sustained through bicycle accidents, suggesting bicycle accidents are a more common cause of clavicle fractures in Sweden than in Scotland. The Scottish study population was however younger than ours, with a mean age of 34 and a lower age limit for inclusion of 13.

As in previous studies, left-sided fractures were slightly more common than right-sided fractures [2, 4, 5, 10], whereas bilateral clavicle fractures and open fractures were uncommon [1, 2, 14].

The distribution of fracture types is consistent with previous results [1–4]. Sociodemographic variations such as age or sports involvement among the population as well as injury mechanism should reasonably affect the distribution, suggesting similarities in these areas between our nationally collected data with previous single-department studies.

Clavicle fractures were the single most common fracture type in polytrauma patients at the Orthopaedic Department, Sahlgrenska University Hospital (Möller M., Sahlgrenska University Hospital 2016, personal communication February 13). The reported frequency of concomitant orthopaedic injuries has varied somewhat between different studies in the past. Robinson [3] reported that among his population of 1 000 patients, 142 required inpatient care, and out of these, 75 had other orthopaedic injuries. A Swedish study [2] reported that only 5% of 185 patients had another extremity fracture with an additional 20% having rib fractures. In comparison to these studies, we had a large proportion of orthopaedic multiple trauma patients in our local population. This observation might be explained with the argument that Uppsala University Hospital is a regional referral centre for polytraumatised patients in need of orthopaedic and neurosurgical treatment.

Our rate of operative treatment was lower than that reported in a meta-analysis of 2 144 midshaft fractures [14], where as many as 47% of the total number of fractures and 79% of the displaced midshaft fractures were treated operatively. The most commonly operated fractures, the displaced midshaft ones, occurred more often in males than in females, which can help explain why the rate of operative treatment was higher in males than in females. In the meta-analysis [14] intramedullary fixation was more common (17%) than in our material (5%). Previous studies have shown similar results with regards to epidemiology and classification of clavicle fractures, which might otherwise have helped to explain the discrepancies in treatment. It therefore seems like treatment decisions are influenced much by local traditions and surgeon preferences, a notion that is supported in the literature [15]. Since convincing evidence to support the selection of one or the other type of treatment (operative versus non-operative treatment, plate fixation versus intramedullary nailing etc.) is missing, this is hardly surprising.

### Strengths of the study

One of the strengths of the present study is that using data from the SFR provides a very large database of clavicle fractures. Data on 2 422 clavicle fractures were uniformly recorded in the SFR according to a pre-specified standard. The material describes national prospective data, reducing the bias of local differences in epidemiology, sociodemographics and treatment traditions. A validation study of the SFR showed high accuracy in classification of tibia fractures when comparing registered data to a gold standard, as well as good inter-observer agreement [22]. Another strength of this observational study is that all data were collected during a recent short period of only 2 years, whereas data collection in earlier studies has often been conducted for many years [1, 3, 13]. This provides an up-to-date overview of epidemiology, classification and treatment of clavicle fractures in recent time. Our minimum age (15 years) was higher than that of many other studies [1, 3, 13], which in our opinion creates a better platform for analysis of an adult population because of the clinically significant high remodelling capacity of clavicle fractures in children and adolescents [23].

### Limitations of the study

The SFR’s coverage at the end of the study period was, with 22 participating departments, approximately 44% of Sweden’s orthopaedic departments. Although more representative of the national population of clavicle fractures than singe-centre studies, the incomplete coverage is a limitation. Completeness has not yet been investigated in the area of clavicle fracture registrations in the SFR. The classification of clavicle fractures in the SFR has not yet been validated.

### Implications and future research

This study is unique in the sense that it assesses uniformly registered data on epidemiology, classification and treatment of clavicle fractures from a large number of orthopaedic departments. The best treatment for clavicle fractures is a debated subject. In order to know how best to treat them, we must first know what and whom we are treating. This study serves as an up-to-date overview of modern clavicle fractures that will hopefully provide a framework for future research on the treatment of clavicle fractures. Future studies should focus on outcome aspects of the treatment of clavicle fractures in order to obtain better guidelines for treatment.

## Conclusions

In conclusion, we have described the epidemiology of clavicle fractures that were registered over a 2-year period in the prospective national SFR for injury mechanism, fracture classification and treatment. The largest patient group was young males. Displaced midshaft fractures were the most common type of clavicle fracture as well as the most frequently operated type of fracture.

## Abbreviations

SFR: The Swedish Fracture Register
